# Novel Biallelic *SQSTM1* Mutation Causing a Subacute‐Onset Complex Movement Disorder with Oculomotor Abnormalities

**DOI:** 10.1002/mdc3.70252

**Published:** 2025-07-29

**Authors:** Ana Luísa de Almeida Marcelino, Nina‐Maria Wilpert, Jonas Leubner, Felix Boschann, Nadja Ehmke, Christoph J. Ploner, Tina Mainka, Markus Schuelke, Andrea A. Kühn

**Affiliations:** ^1^ Department of Neurology with Experimental Neurology, Movement Disorders and Neuromodulation Unit Charité‐Universitätsmedizin Berlin, Corporate member of Freie Universität Berlin and Humboldt‐Universität zu Berlin Berlin Germany; ^2^ Berlin Institute of Health at Charité ‐ Universitätsmedizin Berlin, BIH Biomedical Innovation Academy, BIH Charité (Junior) Clinician Scientist Program Berlin Germany; ^3^ Department of Neuropediatrics Charité‐Universitätsmedizin Berlin, Corporate member of Freie Universität Berlin and Humboldt‐Universität zu Berlin Berlin Germany; ^4^ Center for Chronically Sick Children, Charité‐Universitätsmedizin Berlin, Corporate member of Freie Universität Berlin and Humboldt‐Universität zu Berlin Berlin Germany; ^5^ NeuroCure Cluster of Excellence, Charité‐Universitätsmedizin Berlin, Corporate member of Freie Universität Berlin and Humboldt‐Universität zu Berlin Berlin Germany; ^6^ Institute of Medical Genetics and Human Genetics, Charité‐Universitätsmedizin Berlin, corporate member of Freie Universität Berlin and Humboldt‐Universität zu Berlin Berlin Germany; ^7^ Department of Neurology with Experimental Neurology Campus Virchow Klinikum, Charité‐Universitätsmedizin Berlin, Corporate member of Freie Universität Berlin and Humboldt‐Universität zu Berlin Berlin Germany

**Keywords:** *SQSTM1*, pediatric movement disorders, dystonia, ataxia, oculomotor abnormalities

Biallelic pathogenic variants of *SQSTM1* are known to cause a childhood‐onset syndrome with neurodegeneration, ataxia, dystonia and gaze palsy, abbreviated as “NDAGP.”^1^ Since the first description in 2016, over 30 cases have been reported in the literature. Here, we describe a case of subacute onset “NDAGP” after surgery with general anesthesia in a child with a novel homozygous *SQSTM1* variant and highlight relevant features that can motivate testing for this gene in clinical practice.

## Case Report

**Figure 1:**
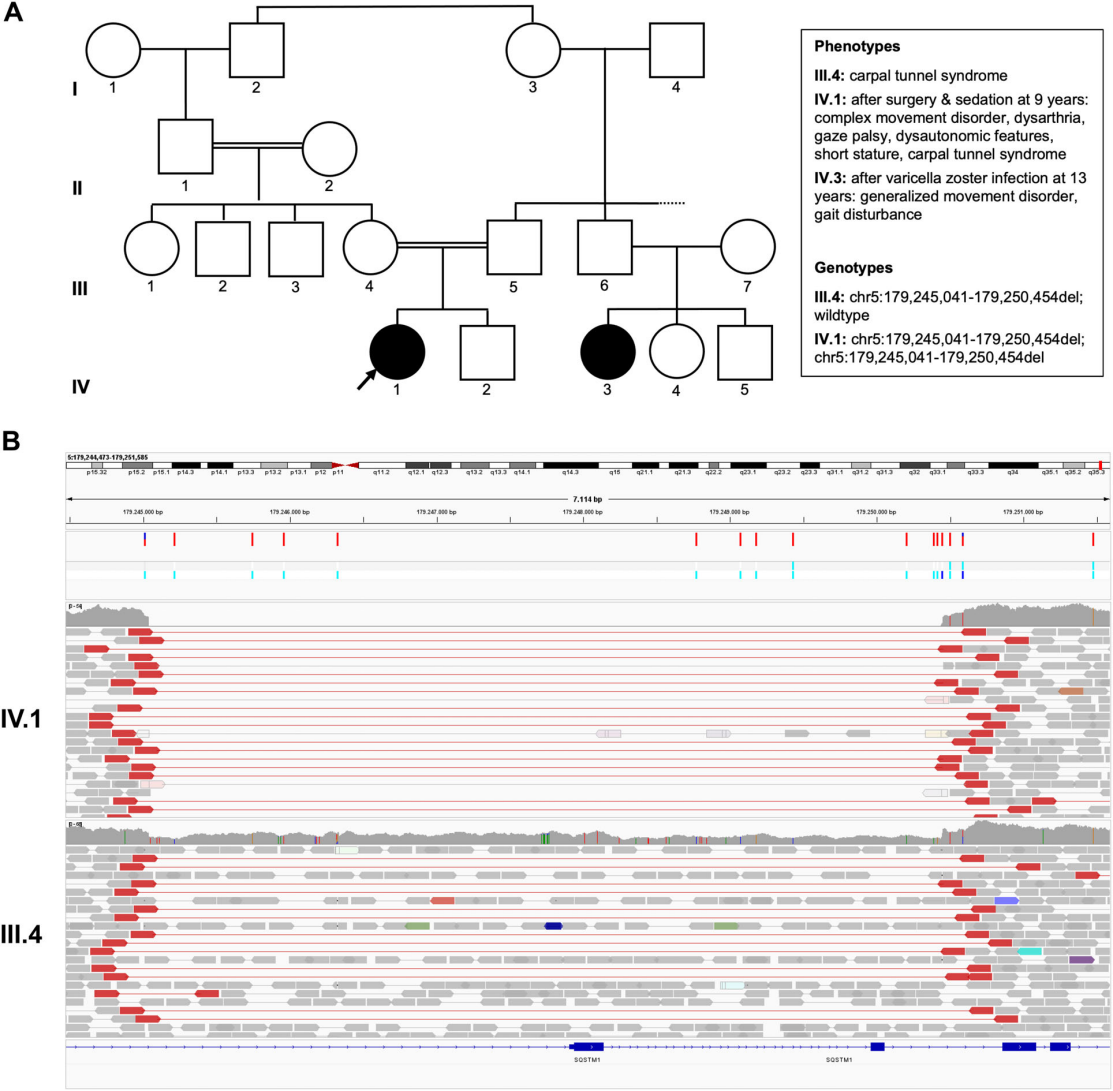
Family pedigree and genetic variant. (A) The index patient is a child of consanguineous parents. Subject III.5 (father) and subject IV.3 were not available for genetic testing. (B) Integrative genome viewer screenshot showing the deletion (NC_000005.9:g.179245041_179250454del) in the homozygous state in the index and in the heterozygous state in the mother. The deletion includes the promoter, 5‐UTR′, exon 1 (including the start codon) and exon 2 of *SQSTM1* (NM_003900.5).

An 18‐year‐old Iranian woman presented at our interdisciplinary pediatric movement disorders outpatient clinic due to a childhood‐onset movement disorder. Pregnancy, birth and global development during childhood were unremarkable. At the age of 9 years, immediately after the third uneventful surgery with general anesthesia in the context of a complex forearm fracture, the parents noticed involuntary grimaces and bilateral hand movements (left > right). Within days to weeks, she developed a generalized movement disorder, slurred speech, impaired fine and gross motor skills as well as an unsteady gait that remained stable thereafter. Her parents were consanguineous, and family history was positive for a generalized movement disorder triggered by a varicella infection in a 13‐year‐old cousin (Fig. 1A).

Clinical examination after moving to Germany at the age of 15 years revealed a complex movement disorder with generalized dystonia, orofacial dyskinesia, choreoathetosis of all limbs, axial hypotonia and cerebellar ataxia (Video 1). Oculomotor examination showed a severe vertical and horizontal gaze palsy with disconjugate saccadic intrusions while attempting smooth pursuit, absent self‐initiated and reflexive saccades, preserved convergence, impaired vestibulo‐ocular reflex in horizontal and absent in vertical plane (Video 2, Transcript in Supplement). Videos were recorded at 18 years of age and the patient had sufficient German knowledge to follow instructions. There were no signs of limb apraxia. Laboratory tests revealed marginally increased antistreptolysin‐antibodies 251 kU/l (reference <200 kU/l) with unremarkable DNase‐B‐antibodies. Further metabolic screening exams in blood and urine, as well as tests for autoimmune, infectious and rheumatologic diseases were unremarkable (Supporting Information). The cerebrospinal fluid revealed isolated oligoclonal bands but was negative for autoimmune antibodies including testing for unknown antigens with indirect immunofluorescence on murine brain slices. Electroencephalogram, echocardiogram, remaining ophthalmological exam and cranial MRIs at 15, 17 and 20 years (Fig. S1) were normal. Incidental findings included a bilateral carpal tunnel syndrome and a genetically confirmed thalassemia. Neuropsychologic testing using a non‐verbal test revealed a low IQ (78). Treatment with methylprednisolone, oral penicillin and levodopa/carbidopa (5.00/1.25 mg/kg/day) led to no improvement. Over the years, treatment with baclofen, trihexyphenidyl and tetrabenazine had no effect on the movement disorder.

**Video 1:** Clinical exam of the index patient. A complex movement disorder including orofacial dyskinesia, generalized dystonia, choreoathetosis is present at rest. With voluntary movement, dystonic posture and movements increase and ataxia becomes evident, especially when examining finger chase and (tandem) gait.

**Video 2:** Oculomotor tests of the index patient. Testing smooth pursuit reveals vertical and horizontal gaze palsy with disconjugate saccadic intrusions. Self‐initiated and reflexive saccades are absent, convergence preserved, vestibulo‐ocular reflex impaired in horizontal and absent in vertical plane.

Duo genome‐sequencing (mother and index patient) was carried out as part of the “Case analysis and decision support” (CADS) research project and the sequence data analyzed using the VarFish software.^2^ Thereby a homozygous deletion (NC_000005.9:g.179245041_179250454del) spanning the promotor region, the 5′‐UTR and exons 1 and 2 of *SQSTM1* (NM_003900.5) (Fig. 1B) was identified. In the gnomAD database there was solely an overlapping small heterozygous deletion (DEL_5_65729) and the variant had not been listed in ClinVar. The deletion was classified as pathogenic according to the ACMG guidelines (PVS1, PM2_sup, PM3_sup, PP4).

## Discussion

*SQSTM1* codes for a scaffold protein that is necessary for ubiquitine‐mediated autophagy and is expressed in various tissues including neurons of the cerebellum and basal ganglia.^3^ Pathogenic heterozygous *SQSTM1* variants can cause a wide variety of phenotypes that reach from amyotrophic lateral sclerosis over myopathy with rimmed vacuoles to Paget's disease and reflect its involvement in multiple cellular processes.^4^ Biallelic pathogenic variants have been shown to cause a childhood‐onset syndrome with neurodegeneration, dystonia, ataxia and gaze palsy.^1^, ^5^ Like our patient, most cases demonstrate predominant ataxia/dystonia combined with other movement disorders and may have different oculomotor abnormalities such as vertical gaze palsy or gaze‐evoked nystagmus. Disease onset typically occurs within the first two decades and cognitive decline or signs of neurodegeneration may only become evident over time (summarized in Table 1).

**TABLE 1:** Clinical and genetic features of previously reported patients with *SQSTM1* variants

Case		*SQSTM1* variant	AAO [y]	Trigger	MRI findings	Movement disorder	Oculomotor abnormalities	Ref.
1–3	F1	c.2 T>A	10–15	nr	Basal ganglia hyperintensity 1/3, basal ganglia iron accumulation 2/3	Dystonia 3/3, parkinsonism 1/3, chorea 1/3, ataxia 2/3	Gaze palsy (vertical) 2/3, gaze‐evoked nystagmus 1/3, saccadic eye pursuit 1/3	^1^
4–6	F2	c.311_312del	10	nr	Mild cerebellar atrophy 3/3	Dystonia 2/3, athetosis 1/3, ataxia 3/3	Gaze palsy (vertical) 3/3, gaze‐evoked nystagmus 1/3	^1^
7	F3	c.286C>T	7	Varicella infection	Mild–moderate cerebellar atrophy	Myoclonus, tremor, ataxia	Oculomotor apraxia	^1^
8,9	F4	c.286C>T	8	nr	‐	Dystonia 1/2, postural instability 2/2, ataxia 2/2	Gaze palsy (vertical) 2/2, gaze‐evoked nystagmus 2/2	^1^
10, 11	F5	c.301 + 2 T>A	6,12	nr	Mild cerebellar atrophy 1/2, enlarged cisterna magna 1/2	Choreoathetosis 2/2, ataxia 2/2	Gaze palsy (vertical) 2/2, nystagmus 2/2	^5^
12, 13	F6	c.934_936delinsTGA	10,12	nr	nr	Dystonia 2/2, choreoathetosis 2/2, ataxia 2/2	Gaze palsy (vertical) 2/2, oculomotor apraxia 2/2, saccadic eye pursuit 2/2	^5^
14–20	F7	c.875_876insT	9–11	nr	nr	Ataxia 7/7	Gaze palsy (vertical) 7/7	^5^
21	F8	c.712_713insTCCTCCGAGTGTGAATTTCCTGA	8	nr	‐	Ataxia	Gaze palsy (vertical), oculomotor apraxia	^10^
22, 23	F9	c.257_259delins35; c.301 + 1G>T (*)	7	nr	Mild cerebellar atrophy 1/2, − 1/2	Dystonia 2/2, ataxia 2/2	nr	^8^
24, 25	F10	c.257_259delins35; c.301 + 1G>T (*)	9,14	TBI, surgery & sedation 1/2	‐ 1/2, encephalomalacia and FLAIR hyperinensities in frontal lobes after TBI 1/2	Dystonia 2/2, chorea 1/2, ataxia 2/2	Supranuclear gaze palsy 2/2, gaze‐evoked nystagmus 1/2	^8^
26	F11	c.823_824delAG	9	nr	‐	Chorea, postural instability, ataxia	Gaze palsy (down), abduction restriction	^11^
27	F12	c.55G>T	6	nr	Mesencephalon, pons hyperintensities	Myoclonus, ataxia	Gaze palsy (vertical & horizontal)	^6^
28	F13	c.790delA	early childhood	nr	nr	Dystonia, ataxia	Oculomotor apraxia	^12^
29	F14	c.838G>T	7	nr	‐	Dystonia, chorea, tremor, ataxia	Gaze palsy (vertical), saccadic eye persuit	^13^
30	F15	g.179825221dupC	10	nr	Mild cerebellar atrophy	Dystonia, ataxia	Gaze palsy (vertical & horizontal), oculomotor apraxia	^7^
31–33	F16	c.1A > G (p. Met1?)	6–7	nr	Mild cerebellar atrophy 1/3	Dystonia 2/3, choreoathetosis 3/3, ataxia 3/3	Gaze palsy (vertical) 3/3, saccadic eye persuit 1/3, nystagmus 1/3	^9^
34, 35	F17	c.1A>G; c.969G>A (*)	3,18	nr	‐	Chorea 2/2, ataxia 2/2	Gaze palsy (vertical & horizontal) 2/2, gaze‐evoked nystagmus 1/2	^14^
36	F18	c.65G>C	7	nr	Mild cerebellar atrophy	Ataxia	Gaze palsy (vertical)	^15^
37	F19	chr5:179,245,041‐179,250,454del	9	Surgery & sedation	‐	Dystonia, choreoathetosis, ataxia	Gaze palsy (vertical & horizontal), impaired VOR, oculomotor apraxia	Current case

*Note*: (*): compound heterozygous mutations (all others homozygous). −, negative/normal; AAO, age at onset; F, family; MRI, magnetic resonance imaging; nr, not reported; TBI, traumatic brain injury; VOR, vestibulo‐ocular reflex. Haack et al^1^, Muto et al^5^, Vethartam et al^10^, Zúñiga‐Ramírez et al^8^, Akkari et al^11^, Kilic et al^6^, Jalali et al^12^, Salari et al^13^, Garg et al^7^, Chacaltana‐Vinas et al^9^, Masuko et al^14^, Mokhtari et al.^15^

New features are gray shaded: (1) in three individuals, the symptoms were preceded by a “trigger,” (2) neuroimaging may be normal, (3) movement disorders are heterogenous, (4) oculomotor abnormalities beyond gaze palsy may exist. Further features summarized from literature also present in this case: dysarthria (30/37); upper motor neuron signs (23/37); dysautonomic features (22/37); short/thin stature (22/35).

Interestingly, the oculomotor disorder in our patient was more severe than in the cases described to date and goes beyond an isolated supranuclear gaze palsy as it could not be overcome by vestibulo‐ocular reflex. Presence of saccadic intrusions in all directions however argues against complete involvement of the final motor pathway for eye movements. The oculomotor disorder suggests a (diffuse) multi‐leveled pathology in the brainstem as recently reported in one patient.^6^ Failure in initiating saccadic and pursuit movements further suggests additional involvement of supratentorial oculomotor structures as in oculomotor apraxia. Clinical manifestation or progression can be accompanied by MRI abnormalities including iron deposition in the basal ganglia or cerebellar atrophy.^1^, ^5^, ^7^, ^8^, ^9^ Nonetheless, our case emphasizes that clinical stability after initial manifestation and normal neuroimaging do not exclude NDAGP.

Functional studies suggest that pathogenic variants in *SQSTM1* lead to an impaired selective autophagy response after cell stress.^1^ Manifestation of the movement disorder triggered by varicella infection (F3:II.1 in,^1^ possibly IV.3 in Figure 1), traumatic brain injury^8^ or multiple surgeries with general anesthesia in our patient may indicate a stress‐dependent pathomechanism and make *SQSTM1* variants a relevant differential diagnosis of other stress‐triggered movement disorders such as *ATP1A3*‐associated dystonia. Additional data will be necessary to support this observation.

Taken together, pathogenic biallelic *SQSTM1* variants lead to a combined (but variable) movement disorder associated with oculomotor abnormalities that can transcend supranuclear gaze palsy. Even though neuroimaging may be normal, subacute manifestation after oxidative stress might raise suspicion for this syndrome and should be investigated in future studies.

## Author Roles

(1) Research project: A. Conception, B. Organization, C. Execution; (2) Statistical Analysis: A. Design, B. Execution, C. Review and Critique; (3) Manuscript Preparation: A. Writing of the first draft, B. Review and Critique.

A.L.A.M.: 1A, 1B, 1C, 3A, 3B;

N.M.W.: 1A, 1B, 1C, 3B;

J.L.: 1C, 3B;

F.B.: 1C, 3B;

N.E.: 1C, 3B;

C.P.: 1C, 3B;

T.M.: 1C, 3B;

M.S.: 1A, 1C, 3B;

A.A.K.: 1A, 1C, 3B.

## Disclosures

**Ethical Compliance Statement:** The authors confirm that the approval of an institutional review board was not required for this work. Written informed patient consent was obtained from the patient to publish the manuscript and the videos. We confirm that we have read the Journal's position on issues involved in ethical publication and affirm that this work is consistent with those guidelines.

**Funding Sources and Conflict of Interest:** ALAM and NMW received a grant from the *Berliner Sparkassenstiftung Medizin* for their collaborative project “Highly specialized care and translational research of pediatric movement disorders.” ALAM and AAK are funded by the Deutsche Forschungsgemeinschaft (DFG, German Research Foundation)—Project‐ID 424778381 – TRR 295. ALAM is a fellow of the Berlin Institute of Health (BIH) Charité Clinician Scientist Program. AAK is supported by the DFG under Germany's Excellence Strategy EXC‐2049 – 390688087 and additionally funded by the Lundbeck Foundation (Grant Nr. R336‐2020‐1035). NMW was supported by the DFG Research Unit 2841 “Beyond the Exome” and is participant in the BIH Charité Junior Clinician Scientist Program for Rare funded by the Charité—Universitätsmedizin Berlin, the Berlin Institute of Health at the Charité (BIH), the Alliance4Rare, and the Berliner Sparkassenstiftung Medizin. FB is a participant in the Clinician Scientist Program (CS4RARE) funded by the Alliance4Rare and associated with the above‐mentioned BIH Charité Clinician Scientist Program. The funders had no role in the design of the study, in the collection, analysis, or interpretation of the data, in the writing of the manuscript, or in the decision to publish the results.

**Financial Disclosures for the Previous 12 Months:** NMW participated in a paid consultancy with Primus Consulting Group GmbH, consulted for the film production company Hellinger‐Doll, and was paid by Biogen for a congress presentation unrelated to this work. AAK has served on advisory boards of Medtronic and has received honoraria and travel support from Medtronic and Boston Scientific outside of this work.

## Supporting information


**Figure S1.** Cranial MRI of index patient. The patient underwent MRI at 15, 17 and 20 years of age. T2‐weighted images of basal ganglia (A), brainstem (B) and cerebellum (C) are shown here at 15 years of age. No abnormalities were found at any timepoint in these and other conventional sequences (T1, FLAIR, susceptibility weighted, DWI, ADC).

**Data S1.** Detailed laboratory work‐up and MRI at age of 15 years.
**Transcript Video 2.** Transcript of German instructions spoken in Video 2.

## Data Availability

Data sharing is not applicable to this article as no new data were created or analyzed in this study.

## References

[1] Haack TBB , Iuso A , Kremer LSS , et al. Absence of the autophagy adaptor SQSTM1/p62 causes childhood‐onset neurodegeneration with ataxia, dystonia, and gaze palsy. Am J Hum Genet 2016;99:735–743. 10.1016/J.AJHG.2016.06.026. 27545679 PMC5010644

[2] Holtgrewe M , Stolpe O , Nieminen M , et al. VarFish: comprehensive DNA variant analysis for diagnostics and research. Nucleic Acids Res 2020;48:W162–W169. 10.1093/NAR/GKAA241. 32338743 PMC7319464

[3] Jakobi AJ , Huber ST , Mortensen SA , et al. Structural basis of p62/SQSTM1 helical filaments and their role in cellular cargo uptake. Nat Commun 2020;111(11):1–15. 10.1038/s41467-020-14343-8. PMC6978347 31974402

[4] Yu CH , Dainton‐Howard H , Mesaros M , Rodriguez‐Porcel F . SQSTM1 mutation presenting as a progressive supranuclear palsy mimic. Mov Disord Clin Pract 2023;10:839–841. 10.1002/MDC3.13707. 37205240 PMC10187011

[5] Muto V , Flex E , Kupchinsky Z , et al. Biallelic SQSTM1 mutations in early‐onset, variably progressive neurodegeneration. Neurology 2018;91(4):E319–E330. 10.1212/WNL.0000000000005869. 29959261 PMC6070386

[6] Kilic MA , Kipoglu O , Coskun O , et al. Homozygous SQSTM1 nonsense variant identified in a patient with brainstem involvement. Brain Dev 2021;43:1039–1043. 10.1016/J.BRAINDEV.2021.06.001. 34147300

[7] Garg D , Kapoor H , Ahmad I , et al. Cognitive impairment, ataxia, dystonia, and gaze palsy due to a novel variant in SQSTM1: new lessons. Mov Disord 2024;39:445–447. 10.1002/MDS.29684. 38279634

[8] Zúñiga‐Ramírez C , de Oliveira LM , Kramis‐Hollands M , et al. Beyond dystonia and ataxia: expanding the phenotype of SQSTM1 mutations. Parkinsonism Relat Disord 2019;62:192–195. 10.1016/J.PARKRELDIS.2018.12.031. 30638816

[9] Chacaltana‐Vinas C , Ramirez‐Pajares P , Manrique‐Palomino A , et al. A novel variant in SQSTM1 gene causing neurodegeneration with ataxia, dystonia, and gaze palsy in a Peruvian family. Mov Disord Clin Pract 2024;11:746–748. 10.1002/MDC3.14025. 38532471 PMC11145125

[10] Vedartham V , Sundaram S , Nair SS , Ganapathy A , Mannan A , Menon R . Homozygous sequestosome 1 (SQSTM1) mutation: a rare cause for childhood‐onset progressive cerebellar ataxia with vertical gaze palsy. Ophthalmic Genet 2019;40(4):376–379. 10.1080/13816810.2019.1666414. 31525130

[11] Akkari M , Kraoua I , Klaa H , et al. SQSTM1 mutation: description of the first Tunisian case and literature review. Mol Genet Genomic Med 2020;8(12) Portico. 10.1002/mgg3.1543. PMC7767559 33135846

[12] Jalali H , Khoshaeen A , Mahdavi MR , Mahdavi M . First report of novel mutation (c.790del) on SQSTM1 gene on a family with childhood onset of progressive cerebellar ataxia with vertical gaze palsy. Clin Case Rep 2022;10(8) Portico. 10.1002/ccr3.6203. PMC9361805 35957775

[13] Salari M , Etemadifar M , Neshat Ghalibaf M , Azizi F , Davoodi M , Asadi S . Neurodegeneration, ataxia, dystonia, and gaze palsy (NADGP) syndrome with nocturnal paroxysmal head tremor. Mov Disord Clin Pract 2023;10(5):835–838. Portico. 10.1002/mdc3.13697. 37205252 PMC10186997

[14] Masuko S , Sato M , Nakamura K , et al. A novel synonymous variant in SQSTM1 causes neurodegeneration with ataxia, dystonia, and gaze palsy revealed by urine‐derived cells‐based functional analysis. Mol Genet Genomic Med 2024;12(11) Portico. 10.1002/mgg3.70044. PMC11588856 39587727

[15] Mokhtari D , Jahanpanah M , Jabbari N , et al. Genetic investigation of patients with autosomal recessive ataxia and identification of two novel variants in the SQSTM1 and SYNE1 genes. Hum Genome Var 2024;11(1). 10.1038/s41439-024-00292-x. PMC11364807 39214971

