# Supplementary material for: Novel Biallelic SQSTM1 Mutation Causing a Subacute‐Onset Complex Movement Disorder with Oculomotor Abnormalities
Source: Mov Disord Clin Pract. 2025 Jul 29;12(11):1995–2000. doi: 10.1002/mdc3.70252 (PMC12999036; doi:10.1002/mdc3.70252)
Supplement: Supplementary file 3 — Figure S1. Cranial MRI of index patient. The patient underwent MRI at 15, 17 and 20 years of age. T2‐weighted images of basal ganglia (A), brainstem (B) and cerebellum (C) are shown here at 15 years of age. No abnormalities were found at any timepoint in these and other conventional sequences (T1, FLAIR, susceptibility weighted, DWI, ADC). [file MDC3-12-1995-s001.pdf]

Supplementary Material for Manuscript

"Novel biallelic SQSTM1 mutation causing a subacute-onset complex movement disorder with oculomotor abnormalities"

Laboratory findings

Hemogram and clinical chemistry
Abnormal (clinically significant): microcytic, hypochromic anaemia with low iron, ferritine, transferrine saturation
Normal or mild change (without clinical meaning): kidney, liver and thyroid function, LDH, differential hemogram, urea, serum uric acid, coagulation parameters

Metabolic
Normal or mild change (without clinical meaning): serum lactate, aminoacids in serum and CSF, acylcarnitines, urine organic acids, copper (24h-urine) and coeruloplasmine, blood ammonia

Auto-immune
CSF: Amphiphysin-Ab, CV2 (CRMP5)-Ab, GAD65-Ab, Hu-Ab, Ma2/Ta-Ab, Ri-Ab, Tr (DNER)-Ab, Yo-Ab, AMPAR1-Ab, GABA-B-Rezeptor-Ab, Glycine recepetor-Ab, LGI1-Ab, Myelin-Ab, metabotrop. Glutamate receptor 5-Ab, AMPAR2-Ab, Contactin-assoc. protein Ab, Dopamin-2-receptor-Ab, DPP-like Protein 6 (DPPX)-Ab, AQP-4-Ab, NMDA-Receptor-Ab

Infectiology
Negative (Serum): HIV, Quantiferone/Tbc, Hepatitis B
Negative (Serum/CSF): Lyme disease, HSV-1, HSV-2 (PCR & serology)

Imaging findings

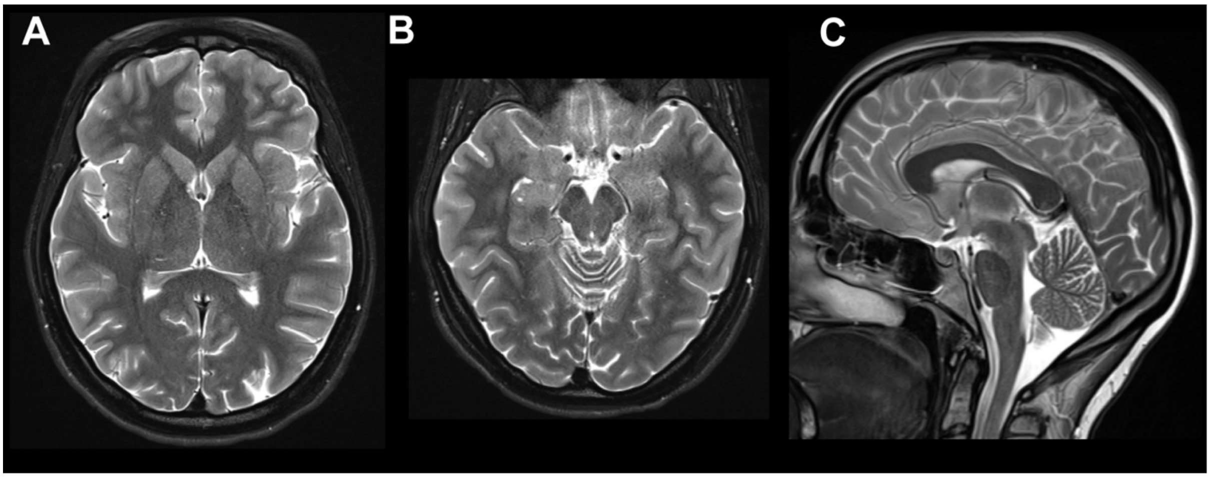

Supplementary Figure 1: Cranial MRI of index patient. The patient underwent MRI at 15, 17 and 20 years of age. T2-weighted images of basal ganglia (A), brainstem (B) and cerebellum (C) are shown here at 15 years of age. No abnormalities were found at any timepoint in these and other conventional sequences (T1, FLAIR, susceptibility weighted, DWI, ADC).
