# Supplementary material for: Novel Biallelic SQSTM1 Mutation Causing a Subacute‐Onset Complex Movement Disorder with Oculomotor Abnormalities
Source: Mov Disord Clin Pract. 2025 Jul 29;12(11):1995–2000. doi: 10.1002/mdc3.70252 (PMC12999036; doi:10.1002/mdc3.70252)
Supplement: Supplementary file 4 — Data S1. Detailed laboratory work‐up and MRI at age of 15 years. Transcript Video 2. Transcript of German instructions spoken in Video 2. [file MDC3-12-1995-s002.docx]

Transcript of Video2_Oculomotor in “**Novel biallelic SQSTM1 mutation causing a subacute-onset complex movement disorder with oculomotor abnormalities”**

Min. 00:53

“Finger” - finger

“Drauf gucken, drauf gucken” – look at it, look at it

“Und zu mir, Kamera” – and back to me, camera

“Finger” - finger

“Und zur Kamera” – and back to the camera
